# Generation of an Oncolytic Herpes Simplex Virus 1 Expressing Human MelanA

**DOI:** 10.3389/fimmu.2019.00002

**Published:** 2019-01-22

**Authors:** Jan B. Boscheinen, Sabrina Thomann, David M. Knipe, Neal DeLuca, Beatrice Schuler-Thurner, Stefanie Gross, Jan Dörrie, Niels Schaft, Christian Bach, Anette Rohrhofer, Melanie Werner-Klein, Barbara Schmidt, Philipp Schuster

**Affiliations:** ^1^Institute of Clinical and Molecular Virology, Friedrich-Alexander-Universität Erlangen-Nürnberg, Erlangen, Germany; ^2^Department of Microbiology and Immunobiology, Harvard Medical School, Boston, MA, United States; ^3^Department of Microbiology and Molecular Genetics, University of Pittsburgh School of Medicine, Pittsburgh, PA, United States; ^4^Department of Dermatology, Universitätsklinikum Erlangen, Friedrich-Alexander-Universität Erlangen-Nürnberg, Erlangen, Germany; ^5^Lab for Immunogenetics, Universitätsklinikum Erlangen, Erlangen, Germany; ^6^Institute of Clinical Microbiology and Hygiene, University Hospital Regensburg, Regensburg, Germany; ^7^Chair of Immunology, Regensburg Center for Interventional Immunology (RCI), University of Regensburg, Regensburg, Germany; ^8^Institute of Medical Microbiology and Hygiene, University of Regensburg, Regensburg, Germany

**Keywords:** vaccine, oncolytic viruses, malignant melanoma, herpes virus, cytotoxic T cell response

## Abstract

Robust anti-tumor immunity requires innate as well as adaptive immune responses. We have shown that plasmacytoid dendritic cells develop killer cell-like activity in melanoma cell cocultures after exposure to the infectious but replication-deficient herpes simplex virus 1 (HSV-1) *d*106S. To combine this innate effect with an enhanced adaptive immune response, the gene encoding human MelanA/MART-1 was inserted into HSV-1 *d*106S via homologous recombination to increase direct expression of this tumor antigen. Infection of Vero cells using this recombinant virus confirmed MelanA expression by Western blotting, flow cytometry, and immunofluorescence. HSV-1 *d*106S-MelanA induced expression of the transgene in fibroblast and melanoma cell lines not naturally expressing MelanA. Infection of a melanoma cell line with CRISPR-Cas9-mediated knockout of MelanA confirmed *de novo* expression of the transgene in the viral context. Dependent on MelanA expression, infected fibroblast and melanoma cell lines induced degranulation of HLA-matched MelanA-specific CD8^+^ T cells, followed by killing of infected cells. To study infection of immune cells, we exposed peripheral blood mononuclear cells and *in vitro*-differentiated macrophages to the parental HSV-1 *d*106S, resulting in expression of the transgene GFP in CD11c^+^ cells and macrophages. These data provide evidence that the application of MelanA-encoding HSV-1 *d*106S could enhance adaptive immune responses and re-direct MelanA-specific CD8^+^ T cells to tumor lesions, which have escaped adaptive immune responses via downregulation of their tumor antigen. Hence, HSV-1 *d*106S-MelanA harbors the potential to induce innate immune responses in conjunction with adaptive anti-tumor responses by CD8^+^ T cells, which should be evaluated in further studies.

## Introduction

Based on the results of a large phase III trial (1), Talimogene laherparepvec (T-VEC) was recently approved as first oncolytic herpes virus 1 for the treatment of patients with stage IIIB, IIIC, or IVM1a malignant melanoma. This attenuated virus induces regression of injected or distant cutaneous, lymphatic, and visceral lesions (2). It preferentially replicates in tumor cells due to defects in the type I interferon pathway, which renders these cells more susceptible to virus replication (3). T-VEC encodes GM-CSF, which contributes to recruitment of antigen-presenting cells to the site of injection. Via lysis of melanoma cells and uptake into antigen-presenting cells, T-VEC enhances cross-presentation of tumor-associated antigens to T cells, which, in particular in combination with immune checkpoint inhibitors, induces strong anti-tumoral responses leading to significantly improved survival of the patients (4, 5).

T-VEC is one of several oncolytic herpes simplex viruses, which have made their way to the clinic. Amongst them are G207, HSV 1716, NV1020, and HF10, which have been used in phase I/II trials in glioma, glioblastoma, melanoma, neuroblastoma, breast, and pancreatic cancer (6). All these viruses are attenuated, but replication-competent. In addition to inactivation of the neurovirulence gene γ34.5 and the TAP-binding protein ICP47, the third generation oncolytic herpes virus Δ47 has mutated the ribonucleotide reductase ICP6, resulting in a more pronounced attenuation of the virus (7).

We have investigated the oncolytic effects of the HSV-1 *d*106S strain, which, in contrast to other oncolytic herpes viruses, is infectious but replication-deficient due to deletions of essential viral genes (8). HSV-1 *d*106S expresses GFP, which can be replaced by other genes of interest via homologous recombination. Using eleven different melanoma cell lines, we have shown that HSV-1 *d*106S is oncolytic, in particular if combined with plasmacytoid dendritic cells (PDC) (9). These cells are major producers of type I interferons (IFN) in the blood upon stimulation with herpes simplex or influenza viruses (10, 11). They surround and occasionally infiltrate primary melanoma lesions and sentinel lymph nodes (12–14).

Due to an immunosuppressive tumor microenvironment, infiltrating PDC are usually immature and tolerogenic, promoting regulatory immunity (15) and tumor progression (16, 17). Upon activation by Toll-like receptor (TLR) agonists, PDC induce a Th1-type immune response and contribute to T cell-mediated tumor regression. In this respect, we have shown that PDC develop strong killer-cell like activity against melanoma cells upon exposure to HSV-1 *d*106S (9). This was similarly observed by others using Toll-like receptor 7 and 9 agonists as well as viral vaccines for stimulation of PDC (18–26).

So far, evidence is accumulating that oncolytic herpes viruses are potent inducers of innate immune responses. Beyond that, robust anti-tumor immunity requires adaptive immune responses. Two recent proof-of-concept trials showed induction of strong (mostly CD4^+^) T cell responses with subsequent delay in reappearance of new metastases in melanoma patients via injection of minigenes coding for different neoantigen-derived peptides (27) or via vaccination using respective peptides in the context of adjuvant (28).

With oncolytic viruses, induction of adaptive immunity is currently based on the uptake and cross-presentation of tumor-specific antigens released from dying tumor cells. To enhance antigen presentation, we envisaged to replace the transgene GFP in HSV-1 *d*106S by the melanoma-associated antigen MelanA/MART-1. We hypothesized that the expression of a tumor antigen in the context of the oncolytic HSV-1 *d*106S may provoke CD8^+^ T cell responses against melanoma cells, combining oncolytic effects of the virus with enhanced expression of melanoma-associated antigens. Hence, such an oncolytic virus may target both innate and adaptive immune responses.

## Materials and Methods

### Cloning of MelanA Into Transfer Plasmid pd27B

The transfer plasmid pd27B containing sequences homologous to HSV-1 *d*106S (8) and a MelanA expression plasmid (29) were propagated in *Escherichia coli* XL-1 Blue cells (Agilent, Böblingen, Germany) and isolated using the PureLink HiPure Plasmid Midiprep kit (Invitrogen/Life Technologies, Darmstadt, Germany). The coding sequence for MelanA was amplified using NheI-MelanA (5′-TAGATAGCTAGCATGCCAAGAGAAGATGCTC-3′) and MelanA-XbaI (5′-GTCCATTCTAGATTAAGGTGAATAAGGTGGTG-3′) (biomers.net, Ulm, Germany). The PCR product and pd27B were digested using NheI and XbaI (NEB, Frankfurt, Germany), followed by dephosphorylation of pd27B. Both products were purified using the QIAquick PCR purification kit (Qiagen, Hilden, Germany), ligated at room temperature overnight, and transformed into XL-1 Blue cells. Correct inserts were identified using T7-EEV-Prom (5′-AAGGCTAGAGTACTTAATACGA-3′; Promega, Mannheim, Germany) with primers 5′-CCGATGAGCAGTAAGACTC-3′; 5′-AGTTGTGGTTTGTCCAAACTC-3′; 5′-TGGATAAAAGTCTTCATGTTGG-3′.

### Cultivation of HSV-1 *d*106S and HSV-1 *d*106S-MelanA

HSV-1 *d*106S is an infectious recombinant strain derived from the HSV-1 *d*106 virus (30). It expresses GFP under the control of a CMV promoter, has a restored susceptibility to aciclovir, and is replication-deficient due to deletions of essential viral genes and promoter regions (8). Complementing E11 cells providing ICP4 and ICP27/47 *in trans* were propagated in DMEM supplemented with 10% heat-inactivated FCS (Sigma-Aldrich, Munich, Germany), 90 U/ml streptomycin, 0.3 mg/ml glutamine, 200 U/ml penicillin, and periodic G418 selection (400 μg/ml). Infected at 90% confluency (MOI 0.1), cells were harvested at 50–60 h when they showed cytopathic effects but were still adherent. After three freeze-thaw cycles, cells were resuspended in DPBS. Supernatants were filtered through 0.45 μm pores and stored at −80°C. The number of infectious HSV-1 particles was quantified using the 50% tissue culture infective dose (TCID_50_) according to the method of Reed and Munch.

### Isolation of HSV-1 *d*106S DNA

Viral DNA was prepared from nucleocapsids following a published protocol (31). Supernatants of infected cell cultures were loaded onto discontinuous OptiPrep gradients (Sigma-Aldrich) of 40% iodixanol overlayed with 20% iodixanol, and subjected to ultracentrifugation using SW41Ti Ultraclear tubes (Beckman Coulter, Krefeld, Germany) at 30,000 rpm for 2 h, without braking at 800 rpm. The visible whitish ring containing viral particles was harvested by side-puncture, transferred to a VTi65 ultraclear tube (Beckman Coulter), and filled with 30% iodixanol, forming a continuous gradient during ultracentrifugation at 55,000 rpm for 6 h. The visible ring was transferred to a SW41Ti ultraclear tube, filled with DPBS, and pelleted at 20,000 rpm for 90 min. Pellets were re-suspended in DPBS, filtered through 0.45 μm pores, and digested using 10×Taq DNA polymerase buffer containing proteinase K (Sigma-Aldrich) and 0.1% (v/v) Tween 20 at 56°C for 1 h. For all subsequent steps, shearing of viral DNA was minimized by cutting off pipet tips and gentle mixing of solutions. The digested pellet was transferred to a phase lock “light” gel tube (5 Prime, Hilden, Germany) and mixed with phenol-chloroform-isoamylalcohol. After centrifugation at 2,000 rpm for 5 min, the aqueous phase was transferred to another phase lock gel tube, recapitulating the step described above. Traces of phenol were eliminated by chloroform extraction. The aqueous solution was precipitated with 7.5 M ammonium acetate and ice-cold ethanol at −80°C overnight. DNA was pelleted at 15,000 rpm at 4°C for 45 min, washed with 70% ethanol, and resuspended in TE-buffer. Purified DNA was not frozen to avoid double-strand breaks. Purity and integrity was checked using NanoDrop UV-spectrophotometry and EcoRI-HF digestion (NEB).

### Homologous Recombination

E11 cells were seeded into 6-well plates to obtain 90% confluency for transfection. pd27B-MelanA was linearized using SwaI (NEB), purified using the QiaQuick PCR purification kit, and mixed with HSV-1 *d*106S DNA at a ratio of 1:4 (w/w) in DMEM plus glutamine. The mixture was heated at 95°C for 3 min, chilled on ice, and mixed with FuGENE HD transfection reagent (Roche, Mannheim, Germany). After incubation at room temperature for 30 min, the mixture was added to E11 cells. Cytopathic effects were identified after 2 days. Non-fluorescent viral plaques were purified using limiting dilution and further analyzed for evidence of homologous recombination.

### Isolation and Cultivation of Cells

PBMC were isolated from EDTA-anticoagulated blood of healthy donors using standard Biocoll density gradient centrifugation (Biochrom AG, Berlin, Germany), as approved by the Ethical Committee of the Medical Faculty, Friedrich-Alexander-Universität Erlangen-Nürnberg (Ref. no. 3299). PBMC were cultivated in RPMI 1640 with supplements described above. For generation of macrophages, PBMC were seeded into Nunc Lab-Tek chamber slides (Thermo Fisher Scientific) and cultivated in the presence of 15% heat-inactivated autologous serum, removing non-adherent cells by trypsin after 3 days. At 10–14 days, macrophages were infected with wild type HSV-1 (32), HSV-1 166v (33), HSV-1 *d*106S, and HSV-1 *d*106S-MelanA. MRC-5 fibroblasts (ATCC® CCL-171^TM^) and melanoma cell lines (IGR-37, IGR-39, ARST-1, ICNI-5li, SK-MEL30, LIWE-7) were cultivated as described (9).

### CRISPR-Cas9 Knockout

The MelanA gene was knocked out from SK-MEL30 cells using CRISPR-Cas9 technology (Addgene, Cambridge, MA). Sequences of single guide (sg) RNAs were taken from the GECKO library (sgMelanA1: 5′-GCACGGCCACTCTTACACCA-3′; sgMelanA2: 5′-TTGAACTTACTCTTCAGCCG-3′) (34) and inserted into LentiCRISPRv2 puro (#52961) (35). Lentiviral stocks were produced from 293T cells transfected with plasmids LentiCRISPRv2 puro, psPAX2 (#12259), and pMD2.G (#12260).

### HLA Typing

High resolution HLA-A, -B, and -C genotyping was performed using the HLA SBT S4 HLA class I kit (Protrans GmbH, Hockenheim, Germany) according to the manufacturer's instructions in full compliance with the HLA typing standards of the European Federation for Immunogenetics (EFI).

### MelanA-Specific T Cell Generation and Coculture

CD8^+^ T cells were purified from PBMC of a HLA-A^*^02:01-positive donor using a CD8 cell isolation kit (Miltenyi Biotec, Bergisch-Gladbach, Germany) and stimulated using artificial antigen-presenting cells (36) loaded with MelanA/MART-1^27L26−34^ peptide (ELAGIGILTV, GenScript, distributed by Biozol, Eching, Germany). The coculture was carried out in M' medium (37) supplemented with 5% autologous plasma and 3% T cell growth factor (38), kindly provided by Mathias Oelke. On day 7 and weekly thereafter until week 4, T cells were restimulated. Purity was assessed using HLA-A^*^02:01/MART-1^27L26−35^ tetramers and found to be > 95%. Coculture with HLA-matched fibroblast and melanoma cell lines was carried out in the presence of Alexa 488-labeled CD107a (eBiosciences/ThermoFisher, Frankfurt, Germany) and Golgi blockers brefeldin A and monensin (Sigma-Aldrich/Merck; 1:1,000) for 4 h. Prior to coculture, melanoma and MRC5 cells were plated at 90% confluency, infected with the respective viruses (MOI 1) for 20 h or loaded with peptide for 1 h, washed, and subsequently overlaid with a total of 1.5 × 10^5^ CD8^+^ T cells in 96-well plates. After FcR blocking, cells were stained with fixable viability dye eFluor 506 (eBiosciences) and anti-CD8 APC/Cy7 (BioLegend, Koblenz, Germany), and, after permeabilization using the BD Cytofix/Cytoperm™ Kit, with anti-IFN-gamma PE-Cy7 or the respective isotype (eBiosciences).

### FACS Analysis

PBMC were exposed to HSV-1 *d*106S and HSV-1 *d*106S-MelanA for 24 h, washed, and incubated with FcR blocking reagent at 4°C for 10 min. Cell populations were stained at 4°C for 20 min, using a published protocol (39) with antibodies to CD3 (Alexa Fluor700; clone UCHT1; BioLegend, London, UK), CD4 (PE-Cy7; clone RPA-T4; BioLegend), CD11c (PE-Cy5; clone B-ly6, BD Biosciences, Heidelberg, Germany), CD14 (APC; clone HCD14; BioLegend), CD19 (APC-H7; clone SJ25C1; BD Biosciences), CD56 (BV-605; clone NCAM16.2; BD Biosciences), and CD304 (PE; clone AD5-17F6; Miltenyi Biotec). Dead cells were stained using PacificBlue (Invitrogen/Life Technologies). Cells were collected using multiparameter LSR-II flow cytometer with FACSDiva software (BD Biosciences) and FCS Express 3 Software (De Novo Software, Los Angeles, CA, USA). MelanA expression was studied in infected cells, using the BD Cytofix/Cytoperm kit with FITC-conjugated (clone A103, Santa Cruz, Heidelberg, Germany) or unconjugated murine anti-MelanA (clone A103, Dako, Hamburg, Germany) and the respective isotype controls followed by Alexa Fluor 555-conjugated goat anti-mouse F(ab′)_2_ (Invitrogen/Life Technologies).

### Western Blot Analysis

Cells were lysed on ice for 30 min (50 mM TRIS, pH 8.0; 150 mM NaCl; 5 mM EDTA; 1% NP-40; 0.1 mM PMSF), heated in sample buffer containing SDS and ß-mercaptoethanol at 105°C for 10 min, separated on a 10% polyacrylamide gel, and transferred to a PVDF membrane (Merck Millipore, Darmstadt, Germany). After blocking with 5% milk powder plus 0.4% Tween 20, samples were incubated with unconjugated MelanA antibody (1:750) at room temperature for 1.5 h or at 4°C overnight, followed by HRP-conjugated rabbit polyclonal anti-mouse IgG (H+L) (DAKO Diagnostics GmbH, Hamburg; 1:1,000) at room temperature for 60 min. After adding ECL solution containing luminol (Sigma-Aldrich) for 1 min, luminescence was recorded using the Fujifilm LAS-1000 plus gel documentation system.

### Immunofluorescence and Confocal Microscopy

E11/Vero cells and macrophages were infected in chamber slides using HSV-1 *d*106S and HSV-1 *d*106S-MelanA (MOI 10) for 16 h, and incubated in DPBS plus 0.3% Triton-X100 at 4°C for 20 min. After blocking in DPBS with 1% BSA (NEB) and 5% FCS, cells were stained with unconjugated anti-MelanA (diluted 1:75 in DPBS plus 1% BSA) at room temperature for 1 h and Alexa Fluor 555-conjugated goat anti-mouse F(ab')_2_ (Invitrogen/Life Technologies, diluted 1:500 in DPBS plus 1% BSA) for 30 min. Slides were washed with DPBS containing DAPI and covered with VectaShield (Vector Laboratories, distributed by Biozol). In some experiments, cell membranes were stained using Alexa Fluor 555-labeled wheat germ agglutinin (5 μg/ml) (Life Technologies). Cells were analyzed using the DMI 6000B inverted microscope and the TCS SP5 laser scanning microscope equipped with the LAS-AF software (Leica Microsystems, Mannheim, Germany).

### Cell Killing Assays

To investigate direct oncolytic effects of HSV-1 *d*106S and HSV-1 *d*106S-MelanA, 1 × 10^4^ melanoma cells were infected with these viruses using different MOI (1 and 10). Cell viability was checked at day 1, 2, 3, and 4 p.i. using the MTT lysis assay according to the manufacturer's recommendations (Trevigen, R&D Systems, Nordenstadt, Germany). Oncolytic effects of MelanA-specific CD8^+^ T-cells were studied in melanoma cells, which had been infected with HSV-1 *d*106S and HSV-1 *d*106S-MelanA (MOI 1) for 8 h, followed by CD8^+^ T-cell coculture (ratio 1:8) for 16 h and subsequent MTT lysis assay. Peptide-loaded cells served as controls.

### Statistics

In our statistical analysis, we used one-way ANOVA for multiple group comparisons with GraphPad Prism version 8. Two-sided *p* < 0.05 were considered significant.

## Results

### Generation of HSV-1 *d*106S-MelanA

**Figure 1:**
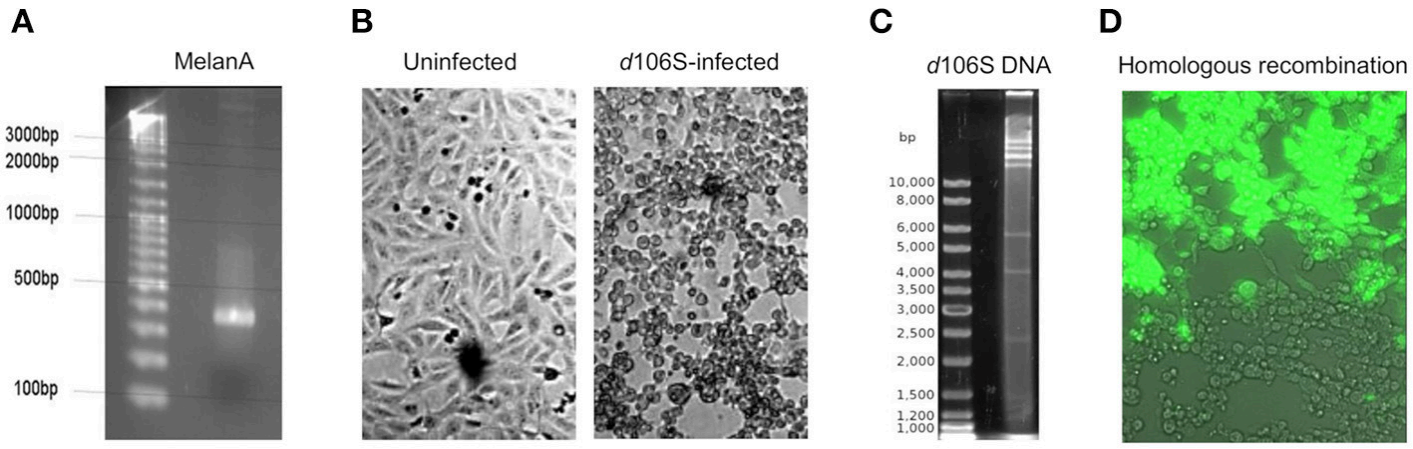
Generation of HSV-1 *d*106S-MelanA. **(A)** Agarose gel image of full-length MelanA amplified from expression plasmid pcDNA3(+) MART-1 (29) using XbaI/NheI-containing primers. **(B)** Light microscopic images of uninfected E11 cells (left) and cytopathic effects induced in these cells 56 h post HSV-1 *d*106S infection (right). **(C)** EcoRI digestion of HSV-1 *d*106S DNA obtained from viral nucleocapsids showing distinct bands as evidence of DNA integrity. **(D)** Overlay of phase contrast and immunofluorescence microscopy of E11 cells harboring fluorescing (upper part) and non-fluorescing (lower part) viral plaques, representing HSV-1 *d*106S and HSV-1 *d*106S-MelanA, respectively, after cotransfection of the linearized transfer plasmid pd27B-MelanA and HSV-1 *d*106S DNA. Light and immunofluorescence microscopy were taken using the DMI 6000B inverted microscope (20 × magnification).

The infectious, but replication-deficient HSV-1 *d*106S expresses GFP under the control of a CMV promoter. To replace this transgene by MelanA, the coding sequence of MelanA was amplified from expression plasmid pcDNA3(+) MART-1 (29) (Figure 1A) and cloned into transfer plasmid pd27B (8). The clone used for homologous recombination was sequenced, revealing identity with the MelanA sequence in GenBank (accession no. NM_005511). After infection of E11 cells with HSV-1 *d*106S (Figure 1B), viral DNA was isolated from nucleocapsids. Purity and integrity of DNA were confirmed by spectrometry and digestion with EcoRI, which revealed distinct bands (Figure 1C). Cotransfection of SwaI-linearized pd27B-MelanA with HSV-1 *d*106S DNA into E11 cells resulted in mostly GFP-expressing (Figure 1D, upper part) and a few non-fluorescent viral plaques (Figure 1D, lower part), which indicated homologous recombination with replacement of GFP in a minority of transfected cells.

### Characterization of HSV-1 *d*106S-MelanA

**Figure 2:**
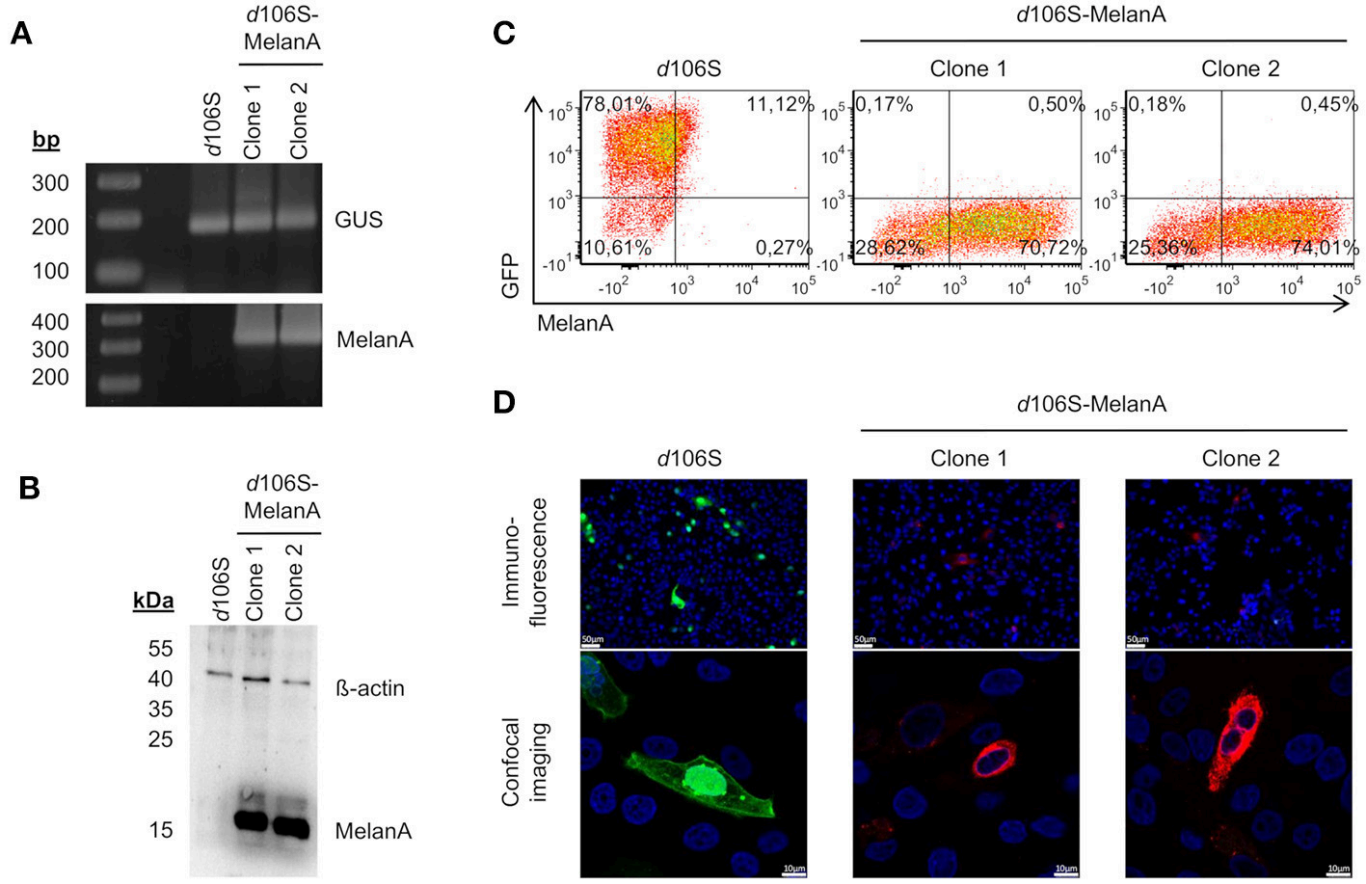
Characterization of HSV-1 *d*106S-MelanA. MelanA expression in E11 cells infected with HSV-1 *d*106S and two non-fluorescent viral clones (MOI 10), which were obtained after cotransfection using HSV-1 *d*106S DNA and transfer plasmid pd27B-MelanA, as evident from **(A)** PCR, **(B)** Western blot, and **(C)** flow cytometry. Controls were **(A)** housekeeping gene ß-glucuronidase (GUS) and **(B)** ß-actin protein. **(D)** Expression of GFP and MelanA in Vero cells infected with HSV-1 *d*106S and two non-fluorescent HSV-1 *d*106S-MelanA clones, analyzed using immunofluorescence microscopy (upper panel) and confocal imaging (lower panel). The immunofluorescence and confocal images were taken using the DMI 6000B inverted microscope (20 × magnification) and the TCS SP5 laser scanning microscope (40 × magnification, 2.5 × zoom), respectively. Scale bars represent 50 and 10 μm in immunofluorescence and confocal images, respectively.

Two non-fluorescent viral plaques were purified via limiting dilution and used to infect E11 cells. The coding sequence of MelanA was detected in cells infected with both clones, but not in cells exposed to HSV-1 *d*106S, while all three infections were positive for the housekeeping ß-glucuronidase gene (Figure 2A). Western blotting detected MelanA protein in E11 cells infected with the two non-fluorescing clones, but not with HSV-1 *d*106S, while ß-actin was present in all three infections (Figure 2B). In flow cytometry, E11 cells infected with HSV-1 *d*106S expressed GFP, while cells infected with the two putative MelanA-expressing clones showed red fluorescence after intracellular staining of MelanA (Figure 2C). Vero cells, which do not support productive HSV-1 *d*106S replication, also expressed GFP or MelanA upon infection (Figure 2D) with mostly nuclear and cytoplasmic expression of GFP and MelanA, respectively, as evident from confocal microscopy. Altogether, we obtained a recombined HSV-1 *d*106S strain expressing MelanA, further termed HSV-1 *d*106S-MelanA.

### Expression of MelanA in Human Fibroblast and Melanoma Cell Lines

**Figure 3:**
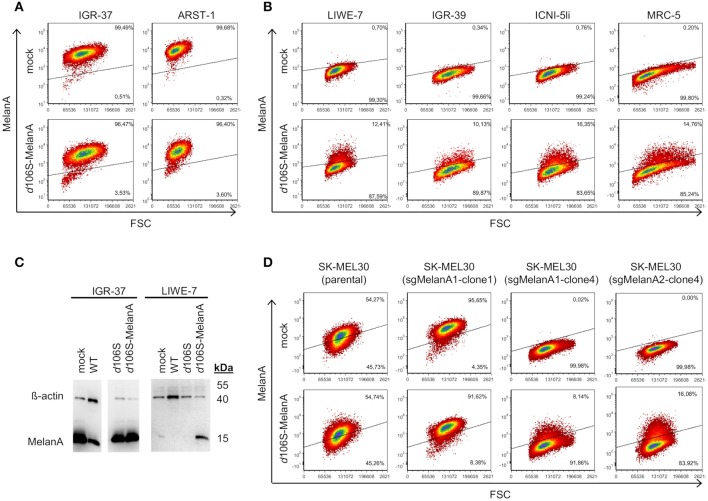
Induction of MelanA expression in melanoma and fibroblast cell lines by HSV-1 *d*106S-MelanA. Expression of MelanA in **(A)** MelanA-positive (IGR-37, ARST-1) or **(B)** MelanA-negative melanoma cells (LIWE-7, IGR-39, ICNI-5li) and in MRC-5 fibroblast cells before (mock) and 20 h p.i. using HSV-1 *d*106S-MelanA. Data are representative of one (MRC-5, ICNI-5li), two (ARST-1, IGR-39), three (IGR-37), and four (LIWE-7) separate experiments. **(C)** Expression of MelanA and ß-actin in melanoma cell lines IGR-37 and LIWE-7, analyzed by Western blotting after infection with HSV-1 wildtype (WT), HSV-1 *d*106S, and HSV-1 *d*106S-MelanA. **(D)** MelanA expression in parental SK-MEL30 cells and after CRISPR-Cas9 treatment resulting in loss of MelanA expression in two of the three analyzed cell clones (sgMelanA1-clone4, sgMelanA2-clone4), while in one cell clone the knockout was not successful (sgMelanA1-clone1). Upon HSV-1 *d*106S-MelanA infection, MelanA was re-expressed in the cell clones, which had lost MelanA expression.

Melanoma cells frequently express MelanA, which may be lost upon immune escape (40). Three of our melanoma cells lines expressed MelanA (IGR-37, ARST-1, SK-MEL30), while three others were negative (LIWE-7, IGR-39, ICNI-5li). We analyzed whether MelanA expression may be restored in the latter upon infection with HSV-1 *d*106S-MelanA (MOI 1). At 20h p.i., IGR-37 and ARST-1 cells still expressed MelanA (Figure 3A), while transgene expression was induced in LIWE-7, IGR-39, and ICNI-5li cells (Figure 3B). Similarly, MelanA expression was induced in MRC-5 fibroblasts. MelanA protein expression was confirmed in IGR-37 and LIWE-7 cells using Western blotting (Figure 3C). These data indicated that melanoma cell lines which did not express MelanA *per se* could be induced to do so.

To exclude upregulation of endogenous MelanA in these cell lines, we used two CRISPR-Cas9 approaches targeting different regions of the MelanA gene (sgMelanA1, sgMelanA2) to knock out this gene in SK-MEL30 cells. Four weeks after lentiviral transduction, MelanA was no longer detectable in 85% of cells. After single-cell sorting, two and five MelanA-negative cell clones were obtained for sgMelanA1 and sgMelanA2, respectively. Upon infection with HSV-1 *d*106S-MelanA, MelanA-negative cell clones (sgMelanA1-clone4, sgMelanA2-clone4) started to re-express MelanA, while MelanA expression was marginally downregulated in parental SK-MEL30 cells and a cell clone with ineffective MelanA knockout (sgMelanA1-clone1) (Figure 3D). These data indicated *de novo* expression of the transgene in the viral context.

### Presentation of MelanA in Human Fibroblast and Melanoma Cell Lines

**Figure 4:**
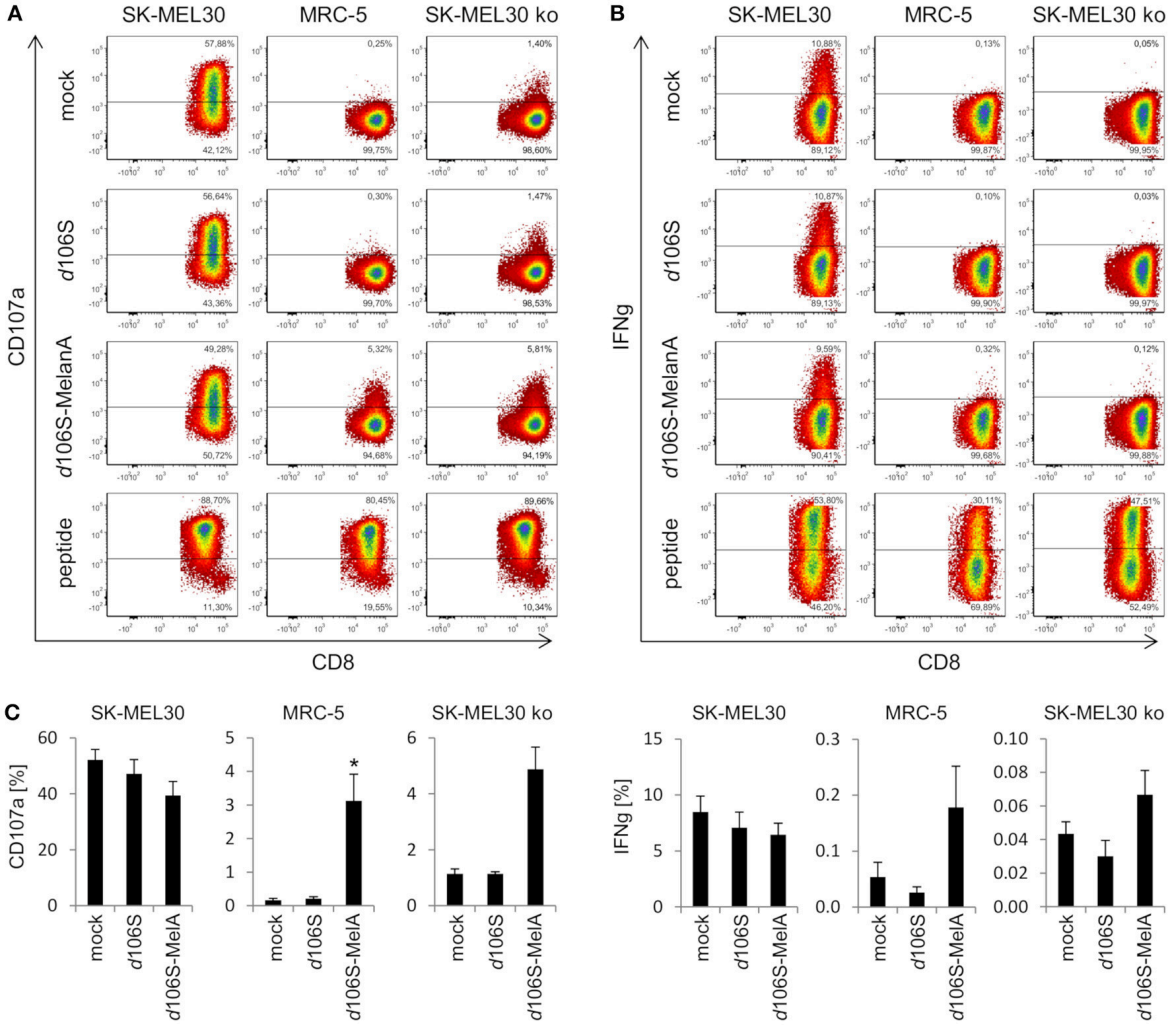
Activation of CD8^+^ T cells by HLA-matched melanoma and fibroblast cell lines infected with HSV-1 *d*106S-MelanA. Degranulation (CD107a) and IFN-gamma (IFNg) production in HLA-A*02:01/MART-1^27L26−34^-specific CD8^+^ T cells after coculture with HLA-matched SK-MEL30 melanoma and MRC-5 fibroblast cell lines for 4 h. In contrast to SK-MEL30 cells, MelanA was not expressed by MRC-5 and SK-MEL30 knockout (ko) cells (sgMelanA1-clone4). Cell lines were infected with HSV-1 *d*106S or HSV-1 *d*106S-MelanA for 20 h prior to coculture or loaded with MelanA peptide MART-1^27L26−34^. T cells were identified as viable CD8^+^ cells after exclusion of doublets. **(A)** One representative experiment and **(B)** mean and standard error of four, five, and **(C)** three separate experiments for SK-MEL30, MRC-5, and SK-MEL30 ko cells, respectively. Percentages of CD107a- and IFNg-expressing cells were compared to mock using one-way ANOVA for multiple group comparisons; ^*^p < 0.05.

In further experiments, we investigated whether expression of MelanA in infected cell lines was followed by presentation of MelanA peptides within the HLA-A context. To this end, we cocultured HLA-A^*^02:01-positive fibroblast (MRC-5) and melanoma (SK-MEL30) cell lines with HLA-A^*^02:01/MART-1^27L26−34^-specific CD8^+^ T cells. As expected, MelanA-expressing SK-MEL30 cells induced CD8^+^ T cell activation after 4 h of coculture, as evident from degranulation (CD107a) (Figure 4A) and IFN-gamma (Figure 4B) production, while MelanA-negative MRC-5 cells failed to do so. Similar results were obtained after infection of cell lines using HSV-1 *d*106S, confirming that virus infection *per se* did not induce CD8^+^ T cell activation. Upon infection of MRC-5 cells with HSV-1 *d*106S-MelanA, however, CD8^+^ T cells showed enhanced surface exposure of CD107a (Figure 4A) and, at least to some extent, IFN-gamma production (Figure 4B). These results indicated processing of virus-encoded MelanA with presentation of the respective peptide in the context of HLA-A in these cells. As a positive control for CD8^+^ T cell activation, MRC-5 and SK-MEL30 cells were exogenously loaded with saturating concentrations of the optimized MelanA/MART-1^27L26−34^ peptide.

To corroborate activation of CD8^+^ T cells by virus-encoded MelanA in melanoma cells, we investigated SK-MEL30 knockout cells. A MelanA-negative cell clone obtained using sgMelanA1 (sgMelanA1-clone4) did not activate HLA-A^*^02:01/MART-1^27L26−34^-specific CD8^+^ T cells, while HSV-1 *d*106S-MelanA infection of this cell clone induced degranulation as evident from the detection of CD107a at the surface of CD8^+^ T cells (Figure 4A). A similar increase in CD8^+^ T cell degranulation was observed after infection of another MelanA-negative SK-MEL30 clone obtained using sgMelanA2 (data not shown). In independent experiments, significant CD8^+^ T cell degranulation was induced by HSV-1 *d*106S-MelanA-infected MRC-5 compared to uninfected cells (0.2% vs. 3.2%, *p* = 0.03) (Figure 4C). A similar trend was observed in SK-MEL30 knockout cells (1.1% vs. 4.9%, *p* = 0.06). Altogether, fibroblast and melanoma cells were induced to express tumor antigen and present respective peptides to tumor antigen-specific HLA-matched CD8^+^ T cells.

### Direct and CD8^+^ T Cell-Mediated Oncolytic Effects of HSV-1 *d*106S-MelanA

**Figure 5:**
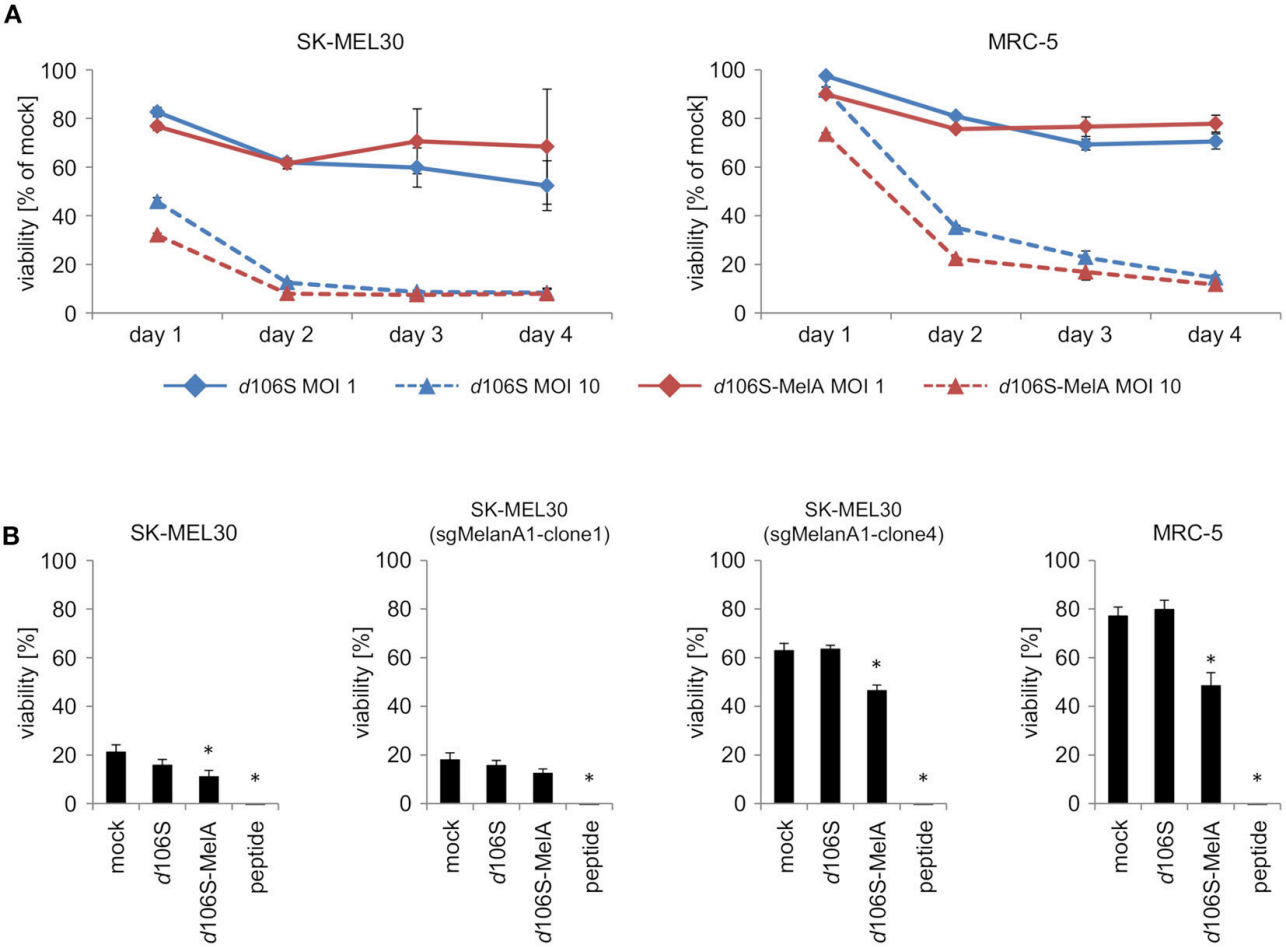
Direct and indirect oncolytic effects of HSV-1 *d*106S-MelanA. **(A)** Viability of SK-MEL30 wild type cells and MRC-5 fibroblast cells was assessed after infection with HSV-1 *d*106S and HSV-1 *d*106S-MelanA (MOI 1 and 10) using MTT lysis assay at day 1, 2, 3, and 4 p.i. **(B)** Parental SK-MEL30 cells and sgMelanA1-clone1, which both expressed MelanA, as well as MelanA-negative sgMelanA1-clone4 and MRC-5 fibroblast cells were infected with HSV-1 *d*106S and HSV-1 *d*106S-MelanA (MOI 1) for 8 h or exposed to MelanA peptide for 1 h prior to coculture with CD8^+^ T cells for another 16 h. MTT values were corrected for values of CD8^+^ T cells only, and normalized to mock-infected cells incubated in the absence of CD8^+^ T cells. Data show mean and standard error of three independent experiments. Viabilities were compared to mock using one-way ANOVA for multiple group comparisons; ^*^p < 0.05.

To investigate direct effects of HSV-1 *d*106S and HSV-1 *d*106S-MelanA on tumor cell killing, SK-MEL30 wild type cells were infected using two different MOI. Oncolytic effects of both viruses on SK-MEL30 cells were comparable. Infection using a MOI of 10 resulted in a significantly stronger reduction of viability than infection using a MOI of 1 (*p* < 0.001 for *d*106S and *p* < 0.01 for *d*106S-MelanA at day 2 p.i.) (Figure 5A). MRC-5 cells were significantly less susceptible to this oncolytic effect (MOI 10) compared to SK-MEL30 cells at day 1 and 2 p.i. (*p* < 0.05).

In further experiments, we studied whether infection of MelanA-negative melanoma cells using HSV-1 *d*106S-MelanA would contribute to the oncolytic effects of MelanA-specific CD8^+^ T cells. SK-MEL30 wild type cells as well as sgMelanA1-clone1, which both expressed MelanA, were readily attacked, while MelanA-negative SK-MEL30 (sgMelanA1-clone 4) and MRC-5 cells remained mostly unaffected (Figure 5B). Exposure of all cell lines to MelanA peptide significantly increased cell death in comparison to untreated cells (*p* < 0.05). Notably, infection with HSV-1 *d*106S-MelanA significantly induced T cell-mediated killing of the MelanA-negative SK-MEL30 (sgMelanA1-clone4) and MRC-5 cells, and even enhanced lysis of the MelanA-expressing SK-MEL30 cell line (*p* < 0.05), whereas infection using HSV-1 *d*106S showed no effect. In sum, HSV-1 *d*106S-MelanA proved to be oncolytic via two effects: direct oncolysis of melanoma cells and induction of enhanced oncolytic activity by MelanA-specific CD8^+^ T cells.

### Expression of the Transgene GFP in Human PBMC and Antigen-Presenting Cells

**Figure 6:**
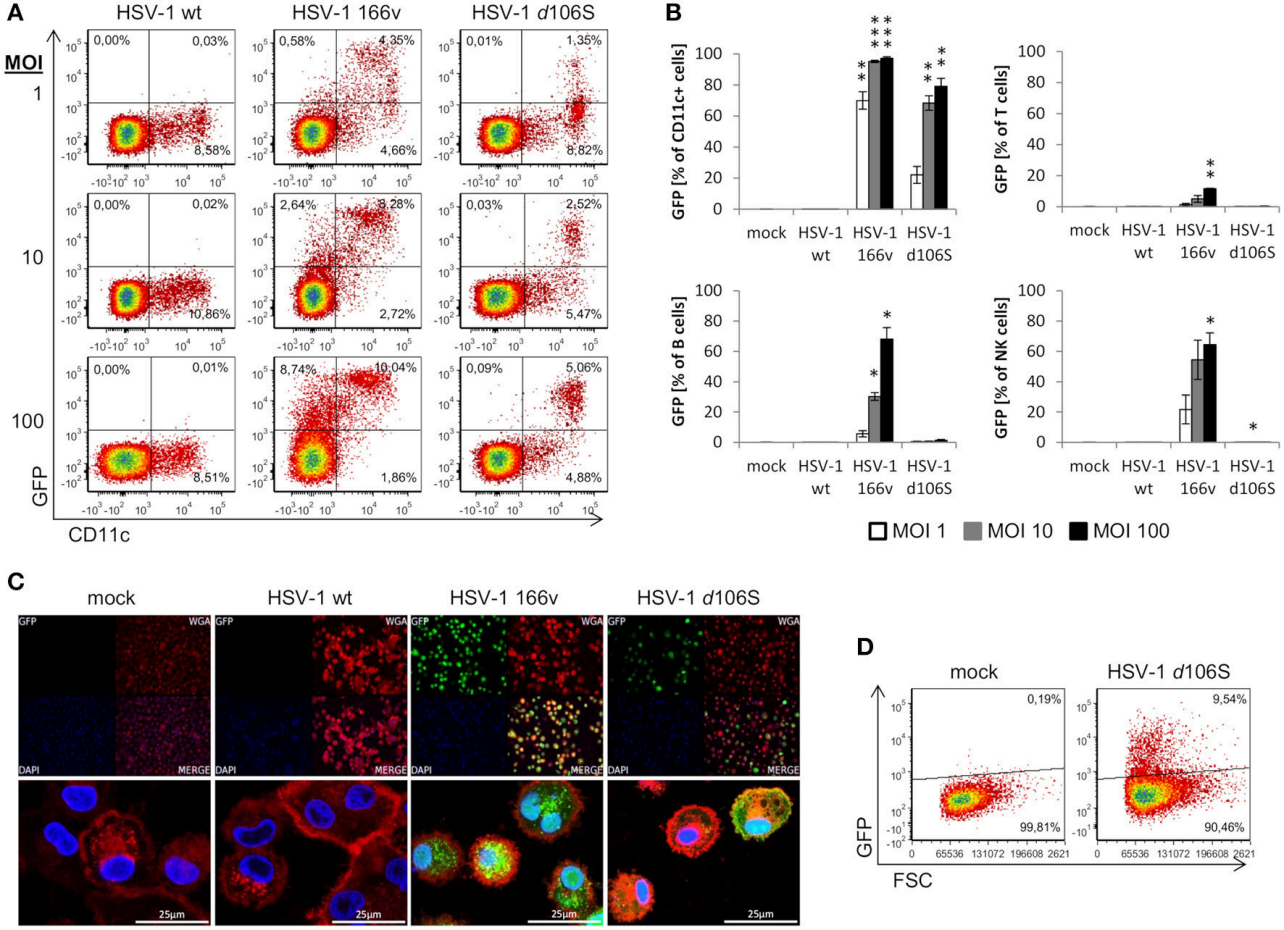
Expression of GFP in human PBMC and macrophages after infection with HSV-1 *d*106S. **(A)** Expression of GFP in CD11c^−^ and CD11c^+^ viable PBMC after infection with HSV-1 wildtype (wt), HSV-1 166v, and HSV-1 *d*106S for 24h, using increasing multiplicities of infection (MOI). One representative experiment out of three is shown. **(B)** Percentage of GFP-expressing cells among CD11c^+^ cells, T cells, B cells, and natural killer (NK) cells. T cells were identified as CD3^+^ cells, B cells as CD3^−^ CD19^+^ cells, NK cells as CD3^−^ CD19^−^ CD56^+^ cells, and CD11c^+^ cells as CD3^−^ CD19^−^ CD11c^+^ cells (for detailed gating strategy see Supplementary Figure 1). Data are shown as mean and standard deviation of three independent experiments using different donors. Expression of GFP in plasmacytoid dendritic cells was not evaluated due to low number of events. Percentages of GFP-expressing cells were compared to mock using one-way ANOVA for multiple group comparisons; ^*^p < 0.05, ^**^p < 0.01, ^***^p < 0.001. **(C)** Expression of GFP in macrophages obtained from a HSV-seronegative donor and exposed to HSV-1 wild type (WT), HSV-1 166v, and HSV-1 *d*106S for 24 h. Cellular membranes were stained using Alexa Fluor 555-labeled wheat germ agglutinin and visualized using confocal imaging. **(D)** Expression of GFP in macrophages exposed to HSV-1 *d*106S for 24 h. Flow cytometry data are representative for two HSV-seronegative donors studied in independent experiments.

We have shown that fibroblast and melanoma cell lines can be induced to express MelanA upon HSV-1 *d*106S-MelanA infection. To find out whether the transgene can also be expressed in antigen-presenting cells, we studied the infection of PBMC obtained from healthy volunteers. Because GFP is more readily detected compared to MelanA, we used HSV-1 *d*106S and focused on monocytes, which can differentiate into antigen-presenting cells. However, CD14 was downregulated at 24 h p.i., as reported previously (41), which precluded proper identification of monocytes. Therefore, antigen-presenting cells including monocytes were labeled using CD11c, which remained expressed at the cell surface. Upon infection with wild type HSV-1, PBMC did not display green fluorescence, while GFP was detected in a proportion of cells exposed to HSV-1 v166 (33) (Figure 6A). This virus codes for a VP22-GFP fusion protein, which is not only expressed but also secreted from infected cells. Therefore, cells with attached fluorescing viruses or VP22 cannot be discriminated from truly infected cells. In contrast, HSV-1 *d*106S expresses GFP under the control of a CMV promotor in infected cells only, and GFP is not incorporated into viral particles. This virus induced GFP expression in CD11c^+^ PBMC, becoming more prominent with increasing MOI (Figure 6A). Individual cell populations were identified as T cells (CD3^+^), B cells (CD3^−^ CD19^+^), NK cells (CD3^−^ CD19^−^ CD56^+^), and CD11c^+^ cells (CD3^−^ CD19^−^ CD11c^+^) using a multicolor flow cytometry panel (Supplementary Figure 1). Again, green fluorescence was not detected upon infection with wild type HSV-1, but upon infection with HSV-1 v166 (CD11c^+^ cells > CD56^+^ NK cells = CD19^+^ B cells > CD3^+^ T cells) (Figure 6B). With HSV-1 *d*106S, GFP was detected in CD11c^+^ cells only, resulting in 22.1, 68.3, and 79.2% of cells infected at MOI of 1, 10, and 100, respectively (Figure 6B).

In addition to primary CD11c^+^ cells, we studied macrophages generated from PBMC of HSV-seronegative donors in the presence of autologous serum. Adherent cells were differentiated into macrophages, which, upon exposure to HSV-1 *d*106S, expressed GFP in confocal imaging (Figure 6C) and flow cytometry (Figure 6D) comparable to the extent observed in fibroblast and melanoma cell lines. Overall, these data indicated expression of virus-encoded GFP in CD11c^+^ cells and in macrophages. We further sought to confirm expression of the transgene upon HSV-1 *d*106S-MelanA infection. However, we were not able to verify MelanA expression in PBMC and macrophages in any of the experimental settings (data not shown).

## Discussion

Oncolytic viruses infect and replicate in tumor tissues. Subsequent lysis of infected cells releases tumor-specific antigens, which are taken up by antigen-presenting cells and induce anti-tumor immune responses via cross-presentation to T cells (42). We sought to optimize the induction of adaptive immune responses by incorporation of a tumor antigen into the viral genome. For this purpose, we used the infectious but replication-deficient HSV-1 *d*106S, which exerts oncolytic activity in particular in combination with PDC (9), and replaced the transgene GFP by MelanA via homologous recombination. Using flow cytometry, Western blotting, and immunofluorescence, protein expression was confirmed in complementing E11 and Vero cells. Upon HSV-1 *d*106S-MelanA infection, we detected transgene expression in MelanA-negative fibroblast and melanoma cells, and in SK-MEL30 cells with specific knockout of the MelanA gene using CRISPR-Cas9 technology. These data confirmed *de novo* expression of MelanA in the viral context.

Subsequent coculture of infected melanoma and fibroblast cell lines with HLA-matched MelanA-specific CD8^+^ T cells verified MelanA-specific activation, as evident from CD8^+^ T cell degranulation upon induced MelanA expression. The infection of parental MelanA-expressing SK-MEL30 cells induced a slightly reduced degranulation of CD8^+^ T cells, most likely due to the oncolytic activity of the virus on target melanoma cells. Notably, we observed an increase after HSV-1 *d*106S-MelanA infection of MelanA-negative cells. It has to be admitted, though, that the degree of IFN-gamma secretion in CD8^+^ T cells was very low. This was not due to a functional limitation of CD8^+^ T cells, as evident from the control using an optimized MelanA peptide. It may rather be the result of the limited MelanA expression induced upon HSV-1 *d*106S-MelanA infection, which was not significantly enhanced using a higher MOI (*data not shown*). The reason may be the efficient oncolytic activity of this replication-deficient virus, resulting in depletion of MelanA-expressing target cells (Figure 5A). Importantly, we observed enhanced CD8^+^ T cell-mediated killing of MelanA-negative melanoma cells upon infection with HSV-1 *d*106S-MelanA, but not upon infection with HSV-1 *d*106S (Figure 5B). These data indicate that HSV-1 *d*106S-MelanA exerts direct and indirect oncolytic effects.

Altogether, our data confirmed that HSV-1 *d*106S-MelanA could re-express MelanA in melanoma cells which have escaped immune recognition via loss of tumor antigen expression. Loss of MelanA expression may be more frequent than previously thought, occurring in three of our 11 melanoma cell lines (Figure 3 and data not shown). As a consequence, MelanA-specific CD8^+^ T cells may be re-directed to infected tumor lesions, which will become re-accessible to this adaptive CD8^+^ T cell response. In this case, an efficient oncolysis will be mediated by HSV-1 *d*106S-MelanA as well as by innate and adaptive immune cells. The apoptotic and necrotic tumor cells will serve as source for new tumor-associated antigens, which have evolved during tumor progression. In this respect, it has previously been shown that apoptotic cells, which were killed by infection with replication-deficient HSV, served as vaccines by pulsing DCs (43). Apoptotic debris will be phagocytosed by dendritic cells and cross-presented to T cells. In this respect, CD11c^+^ cells and macrophages may also play an important role.

Our tumor vaccine may profit from incorporating other tumor antigens which are targets of cytotoxic CD8^+^ T cells, like the MAGE-A family, tyrosinase, NY-ESO1, gp100 or neoantigens (44). With the incorporation of MelanA into HSV-1 *d*106S, however, viral stocks harbored slightly less infectious virions compared to the parental strain. The generation of infectious stocks may become increasingly challenging with the incorporation of additional tumor-associated antigens. The difficulty in inserting full-length sequences of tumor antigens may be overcome by introducing much smaller genomic information as minigenes coding for tumor antigen-derived peptides (27). It may also be worth cloning the coding sequences of tumor antigens or peptides into T-VEC, which is fully replicative and thus more virulent than HSV-1 *d*106S-MelanA.

The minor virulence of the non-replicative HSV-1 *d*106S-MelanA in comparison to the replication-competent T-VEC strain may be advantageous for the infection of antigen-presenting cells: a reduced cytotoxicity may facilitate the presentation of tumor peptides in the context of HLA-ABC. For these purposes, we infected PBMC with HSV-1 *d*106S, showing GFP expression in CD11c^+^ antigen-presenting cells, but not in other immune cells. Subsequently, we noticed expression of GFP in macrophages comparable to the extent of MelanA expression in infected MRC-5 cells. However, we were not able to detect MelanA expression in any of the immune cells investigated, which was unexpected because both transgenes are expressed from the same CMV promoter. So far, it is unclear whether expression of MelanA in antigen-presenting cells is too low to be detected reliably, or whether MelanA is proteasomally degraded and presented on HLA-ABC immediately after mRNA translation.

More importantly, HSV-1 *d*106S has been shown to induce CD8^+^ T cell responses *in vivo*. To this end, studies in mice and monkeys (45–47) revealed that HSV-1 *d*106S can not only activate, but also induce virus-specific CD8^+^ T cells. This *de novo* induction may be more difficult with tumor-associated antigens (with the exception of neoantigens), which, as autoantigens, need to overcome self-tolerance. *De novo* induction can occur via direct presentation of the tumor antigen synthesized in the cytosol or via indirect cross-presentation after endocytosis of the tumor antigen, export into the cytosol and proteasomal degradation, transport to the endoplasmic reticulum and loading on HLA-ABC. Whether the vaccine HSV-1 *d*106S-MelanA can induce expansion of MelanA-specific CD8^+^ T cells, and if so, which of the two mechanisms hold true, needs to be evaluated in further studies. It would be particularly valuable to study these effects *in vivo* using suitable animal models. The immune stimulation following intratumoral injection of the oncolytic virus *in vivo* may enhance the CMV promotor activity and thus contribute to a more efficient transgene expression.

A further prospect of our research is the combination of oncolytic viruses with other anti-cancer approaches like checkpoint inhibitors, chemotherapy, targeted therapy, and radiation therapy (42, 48, 49). It may even be interesting to test oncolytic viruses in combination with tumor-specific peptides. These conditions may reduce the immune-inhibitory activities of tumors and help tumor antigen-specific CD8^+^ T cells to gain access to the malignant lesion. In addition, a new generation of oncolytic herpes viruses has been designed, which is less virulent due to deletion of ICP6 in addition to inactivation of neurovirulence factor γ34.5 and antagonist of the host cell's transporter associated with antigen presentation, ICP47. This oncolytic herpes virus allows a broader applicability and is currently being tested in glioblastoma and prostate cancer patients (7).

For these reasons, our approach to develop an oncolytic herpes virus which augments antitumor responses by coding for a tumor antigen appears to be promising for further combination immunotherapies against malignant melanoma. It may also be promising for other tumors. This may be true in particular for tumors which are known to be infiltrated by PDC, like head and neck squamous cell carcinoma (50), and ovarian (51, 52) and breast cancer (53, 54). A tumor antigen-expressing HSV-1 *d*106S may target both PDC and myeloid dendritic cells, which cooperate in inducing effective anti-tumor T cell responses (55).

## Author Contributions

JB, MW-K, DK, BS, and PS conceived the experiments. JB, ST, AR, MW-K, BS, and PS performed the experiments, analyzed and interpreted the data. DK and ND provided the parental HSV-1 *d*106S virus, transfer plasmid, and complementing cell line. BS-T and SG provided melanoma cell lines. JD and NS contributed the MelanA expression plasmid. MW-K contributed MelanA-specific CD8^+^ T cells to the project. CB performed HLA-typing of cell lines and donors, and BS and PS wrote the manuscript. All authors were involved in critically reading of the manuscript and approved the final version of the manuscript.

### Conflict of Interest Statement

DK is a co-inventor on patents held by Harvard University that includes claims on the use of replication-defective mutant viruses such as *d*106S vaccine vector and immunomodulatory agent. The remaining authors declare that the research was conducted in the absence of any commercial or financial relationships that could be construed as a potential conflict of interest.

## Abbreviations

HSV: Herpes simplex virus
IFN: interferon
ko: knockout
MOI: multiplicity of infection
PBMC: peripheral blood mononuclear cells
PCR: polymerase chain reaction
PDC: plasmacytoid dendritic cells
p.i.: post infection
sg: single guide.
