# Supplementary material for: Generation of an Oncolytic Herpes Simplex Virus 1 Expressing Human MelanA
Source: Front Immunol. 2019 Jan 22;10:2. doi: 10.3389/fimmu.2019.00002 (PMC6349778; doi:10.3389/fimmu.2019.00002)
Supplement: Supplementary file 1 [file Image_1.pdf]

Supplementary Material

Generation of an Oncolytic Herpes Simplex Virus 1 Expressing Human MelanA

Jan B. Boscheinen, Sabrina Thomann, David M. Knipe, Neal DeLuca, Beatrice Schuler-Thurner, Stefanie Gross, Jan Dörrie, Niels Schaft, Christian Bach, Melanie Werner-Klein, Anette Rohrhofer, Barbara Schmidt*, and Philipp Schuster

***Correspondence:** Barbara Schmidt, barbara.schmidt@ukr.de

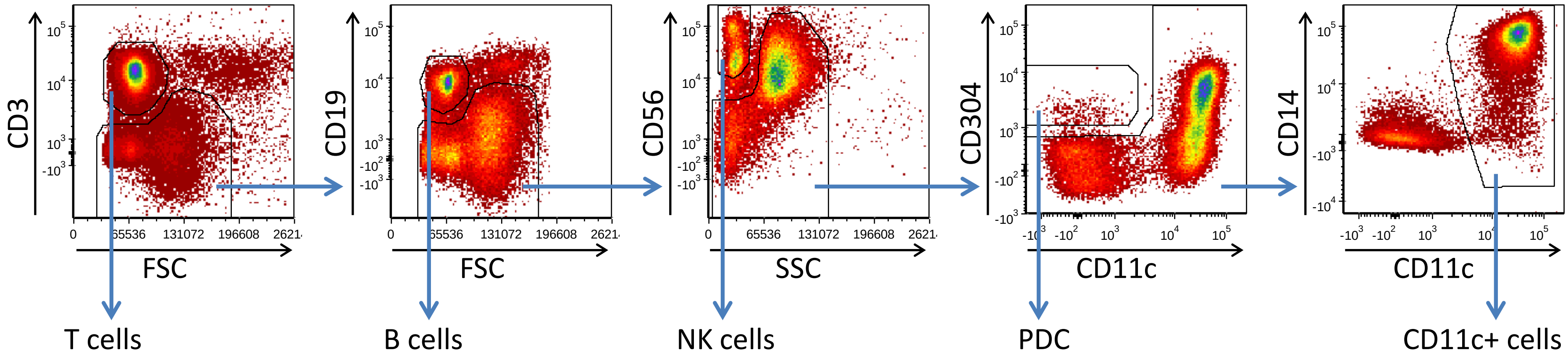

Supplementary Figure 1. Identification of cell populations within PBMC using a multi-color flow cytometry panel, modified from a published protocol (Maecker et al., 2012). After life/dead cell discrimination using PacificBlue staining, T and B cells were identified based on size and expression of CD3 or CD19. Subsequently, natural killer (NK) cells were determined based on granularity and staining for CD56. Plasmacytoid dendritic cells (PDC) were identified as CD11c$^-$ CD304$^+$ cells. The remaining CD11c$^+$ cell population does neither contain B, T, and NK cells nor PDC and mostly consists of CD14$^+$ monocytes.
